# Time-feature attention-based convolutional auto-encoder for flight feature extraction

**DOI:** 10.1038/s41598-023-41295-y

**Published:** 2023-08-30

**Authors:** Qixin Wang, Kun Qin, Binbin Lu, Huabo Sun, Ping Shu

**Affiliations:** 1 School of Remote Sensing and Information Engineering, Wuhan University, Wuhan, 430079 China; 2 Engineering and Technical Research Center of Civil Aviation Safety Analysis and Prevention, China Academy of Civil Aviation Science and Technology, Beijing, 100028 China

**Keywords:** Aerospace engineering, Information theory and computation

## Abstract

Quick Access Recorders (QARs) provide an important data source for Flight Operation Quality Assurance (FOQA) and flight safety. It is generally characterized by large volume, high-dimensionality and high frequency, and these features result in extreme complexities and uncertainties in its usage and comprehension. In this study, we proposed a Time-Feature Attention (TFA)-based Convolutional Auto-Encoder (TFA-CAE) network model to extract essential flight features from QAR data. As a case study, we used the QAR data landing at the Kunming Changshui International Airport and Lhasa Gonggar International Airport as the experimental data. The results show that (1) the TFA-CAE model performs the best in extracting representative flight features in comparison to some traditional or similar approaches, such as Principal Component Analysis (PCA), Convolutional Auto-Encoder (CAE), Self-Attention-based CAE (SA-CAE), Gate Recurrent Unit based Auto-Encoder (GRU-AE) and TFA-GRU-AE models; (2) flight patterns corresponding to different runways can be recognized; and (3) anomalous flights can effectively deviate from many observations. Overall, the TFA-CAE model provides a well-established technique for further usage of QAR data, such as flight risk detection or FOQA.

## Introduction

Civil aviation is constantly striving to improve flight safety. To change the accident-prone nature of Chinese civil aviation and improve flight safety, the Civil Aviation Administration of China (CAAC) decided in 1997 to make the flight quality monitoring project mandatory for all transport airlines. By January 1, 1998, all transport aircraft registered in China were required to install a Quick Access Recorder (QAR) or equivalent equipment to record all aircraft flight status during flights. The flight data recorded by QARs are used to monitor flight operations, aircraft performance, etc., to detect faulty flights that deviate from standard flight procedures. Furthermore, the causes of faulty flights are analyzed and then addressed by developing corresponding improved guidance measures, resulting in further flight quality improvements. At the end of 2013, the flight Quality Monitoring Base Station Construction Project was approved by the CAAC to collect, process and analyze all the QAR data of aircraft in China. By the end of 2017, the base station had collected QAR data from more than 3000 aircraft taken by all 51 transport air carriers in China's civil aviation category. A huge amount of rich flight data is continuously gathered at this base station, which provides a complete database for studying flight risks as well as data-driven methods.

Automatic Dependent Surveillance Broadcast (ADS-B) data is another kind of flight data and also used for flight quality monitoring, such as the aircraft landing time^1^, estimation of aircraft arrival time^2,3^. ADS-B data has a good timeliness compared to QAR data, but only a limited number of flight parameters (about 40 flight parameters) are recorded, which makes it insufficient to be used in complex application scenarios. Compared to ADS-B data, QAR data are typically featured by high-dimensional and high-frequency data to record details of a flight, including time, position, flight operations, flight attitude, flight dynamics and the external environment (up to 2000 flight parameters). The recorded Flight parameters reflect the status of aircraft’s system. For example, excessive value of flight parameter *AOA (Angle of attack)* may suggest potential risks of aircraft stalling while the flight parameter *VRTG (Normal acceleration)* is usually used to indicate the heavy landing when an aircraft touches the ground. Therefore, the QAR data can be used to monitor and detect various flight events. However, these features result in extreme complexities and uncertainties in its usage and comprehension. Feature extraction, as a typical data mining topic, provides a technical means to solve the dimensional curse^4^. It plays a key role in many applications, such as classification^5^, regression^6^, and data mining^7,8^, and is also a prior basis in fault diagnosis that focuses on fault feature extraction^9–12^.

Principal Component Analysis (PCA), by which raw data are projected onto their principal dimensions according to the variance-covariances of the original samples^13^, is the most commonly used unsupervised method for feature extraction^14,15^. Linear Discriminant Analysis (LDA) and its variant, Marginal Fisher Analysis (MFA), are two supervised feature extraction methods, among which LDA finds a useful linear subspace by optimizing discriminant class data^16^ and MFA characterizes the interclass separability and intraclass compactness of the given data to obtain the optimal projection^17^. All the above feature extraction methods have the same shortcoming: all the projections are linear transformations. Although other studies^18–20^ have attempted to solve this problem using nonlinear kernel functions, the features extracted by the developed approaches may fail to cover all useful information of the input raw data since diverse nonlinear correlations exist in the complex industrial data^21^.

**Figure 1:**
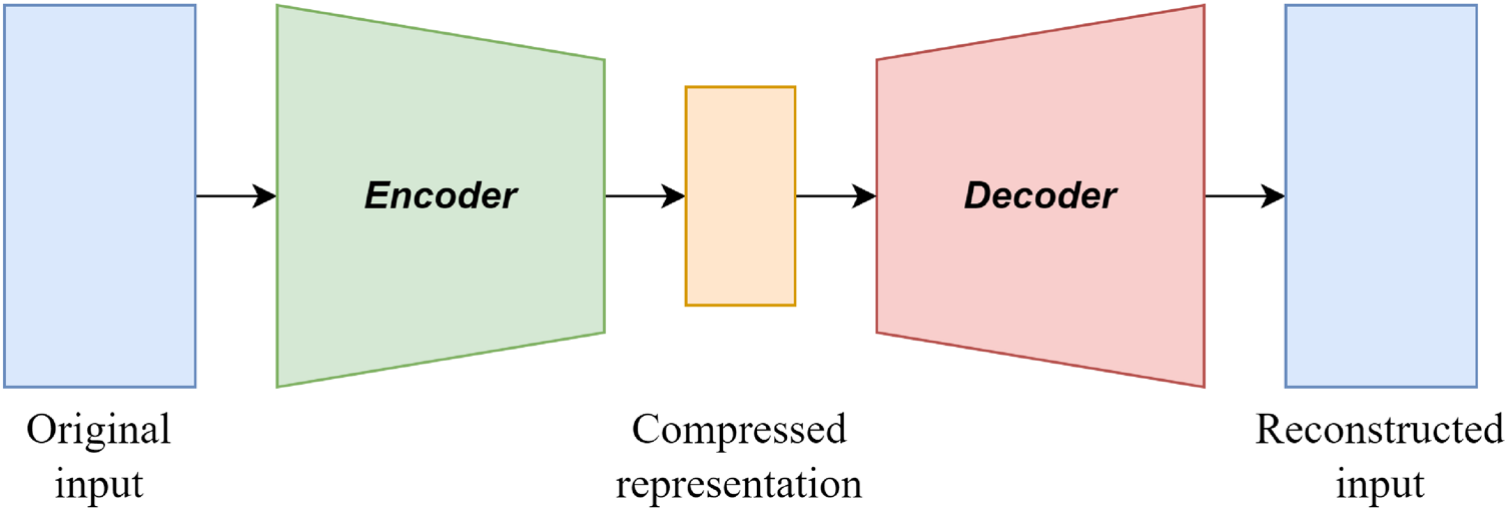
The pictorial representation of an autoencoder network model.

With the continuous development of Artificial Neural Networks (ANNs), they have become powerful technologies for approximating complicated functions and have achieved great success across various industrial applications. An Auto-Encoder (AE), containing an encoder and a decoder, is a special ANN model that extracts features by minimizing the reconstruction error in an unsupervised manner. The original input data are first mapped into a low-dimensional representation space to obtain the most appropriate features; the decoder then maps the features in the low-dimensional representation space to the input space. The loss error between the original input of the encoder and output of the decoder is used as the loss error to train the resulting model. Figure 1 shows a pictorial representation of the autoencoder network model.

AEs and their variants^22–24^ have been applied in various fields, such as fault diagnosis^25,26^, smart grids^27^, and Natural Language Processing (NLP)^28^. However, the features extracted by the traditional AE may fail to satisfy the final discrimination task^29^. For multi-feature time series data, the traditional AE directly maps the original input to learn features, but this process ignores the inter-time and inter-feature relationships. In addition, previously developed feature extraction methods are not based on the requirements of specific applications, resulting in extracted features that are not applicable to realistic application tasks. In this article, a Time Feature Attention (TFA) module is developed to capture the internal relationship between different flight moments as well as the internal relationship between different flight parameters. On this basis, a TFA-based Convolutional AE (TFA-CAE) is proposed to perform feature extraction of QAR flight time series data. The remainder of this paper is organized as follows. The methodology used in our research is presented in "Data and methodology" section, where the details of the TFA and TFA-CAE are described in "TFA module" and "TFA-CAE model for QAR feature extraction" sections, respectively. "Case study" section presents the experimental results of a case study. This study is summarized in "Conclusion and discussion" section.

## Data and methodology

### QAR data processing

During flight, aircraft are generally influenced by various kinds of factors, such as the external meteorological environment (speed and direction of wind, temperature and atmospheric pressure, etc.), conditions of the aircraft itself (status of engine, flight control settings, etc.), competencies and pilot techniques. The complex impacts of these factors on the aircraft are constant and fluctuate throughout the flight^30^. Although these factors are always in flux, their impacts on the aircraft are eventually transformed into changes in the kinematic and attitude flight parameters of the aircraft^31^. Thus, we select the attitude and kinematic flight parameters to perform feature extraction. The details of the flight parameters used in this article are shown in Table 1.

**Table 1:** Details of the selected flight parameters.

Name	Parameter name in the QAR	Units
Angle of attack	AOA	deg
Angle of pitch	PITCH	deg
Angle of roll	ROLL	deg
Angle of flight path	FPA	deg
Head angle (magnetic north)	HEAD_MAG	deg
Rate of pitch change	PITCH_RATE	deg/s
Indicated air speed of calibration	IASC	knot/s
Instantaneous vertical velocity	IVV	g
Lateral acceleration G-force	LATG	g
Longitudinal acceleration G-force	LONG	g
Vertical acceleration G-force	VRTG	g
Radar altitude of calibration	RALTC	ft

**Figure 2:**
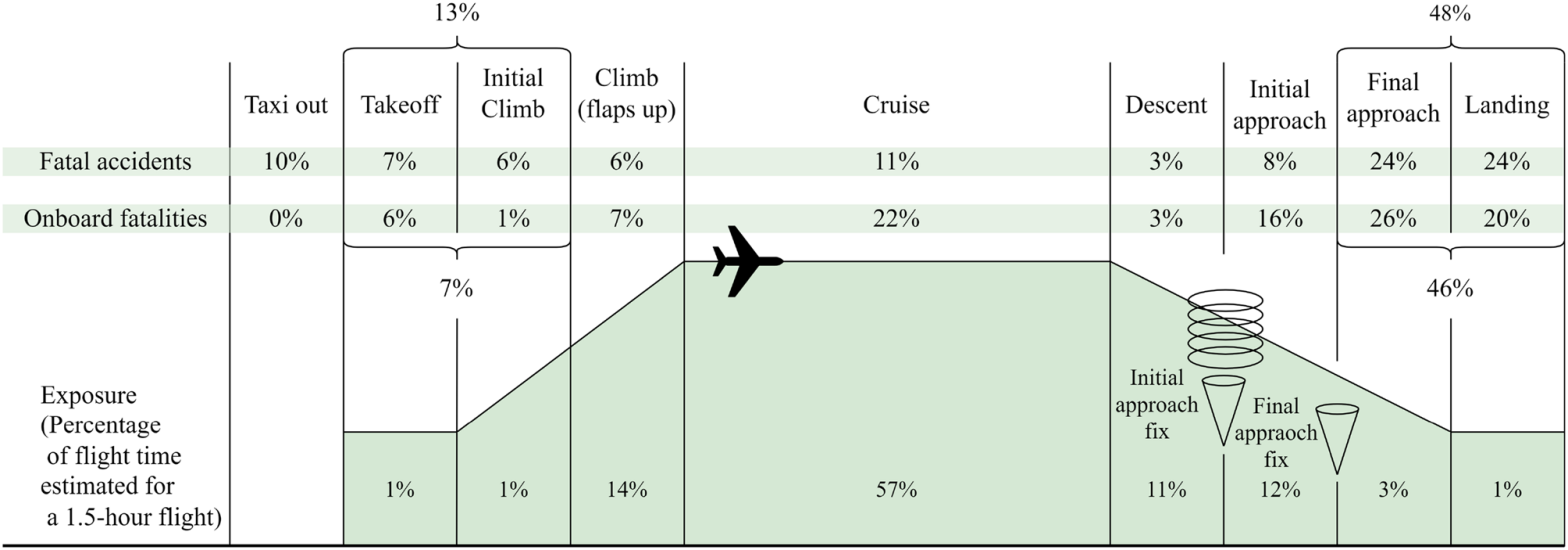
Percentages of fatal accidents and onboard fatalities by phase of flight from 2008 to 2017^32^.

Figure 2 shows the fatal accidents and onboard fatalities in each flight phase from 2008 to 2017^32^. From the statistical results, we can see that the landing phase occupied only 1% of the total flight time but yielded high percentages of fatal accidents and onboard fatalities (up to 24% and 20%, respectively). Therefore, the landing phase is the focus of this article.

**Figure 3:**
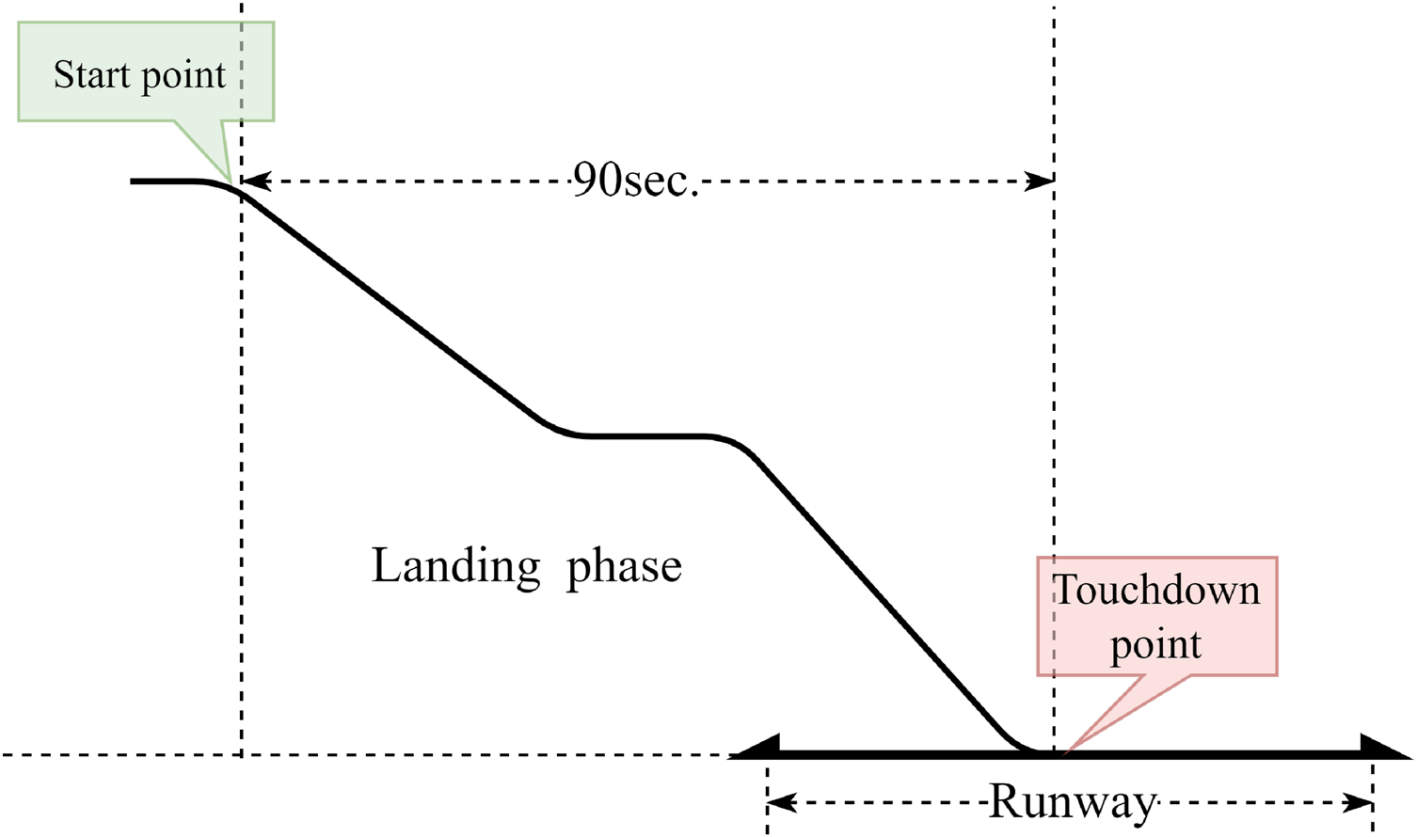
Schematic diagram of the data sampling process during the landing phases.

The specific study flight phase focused on in our research is illustrated in Fig. 3. Since landing phases occupy approximately 90 s in duration, as shown in Fig. 2, a sample duration of 90 s is used in this flight phase. Specifically, we start sampling at 90 s before the touchdown point and end sampling at the touchdown point. For each sampling moment, the values sampled are all the flight parameters shown in Table 1.

### TFA module

The function of the attention mechanism has been widely demonstrated in many previous studies^33–40^. The attention mechanism, on the one hand, helps a model to know the key places to focus on and, on the other hand, enhances the representational value of interests^40^. For a given QAR time series data in this article, we aim to specify when (the key time of the QAR data) and which (the key flight parameters of the QAR data) to focus on and to simultaneously enhance their corresponding representational values with the employment of an attention mechanism. In this article, we propose a TFA module to exploit both time and feature attention based on an efficient architecture.

**Figure 4:**
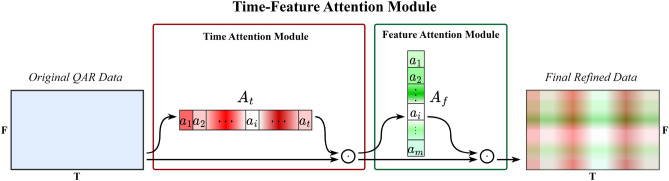
Overview of the TFA module.

**Figure 5:**
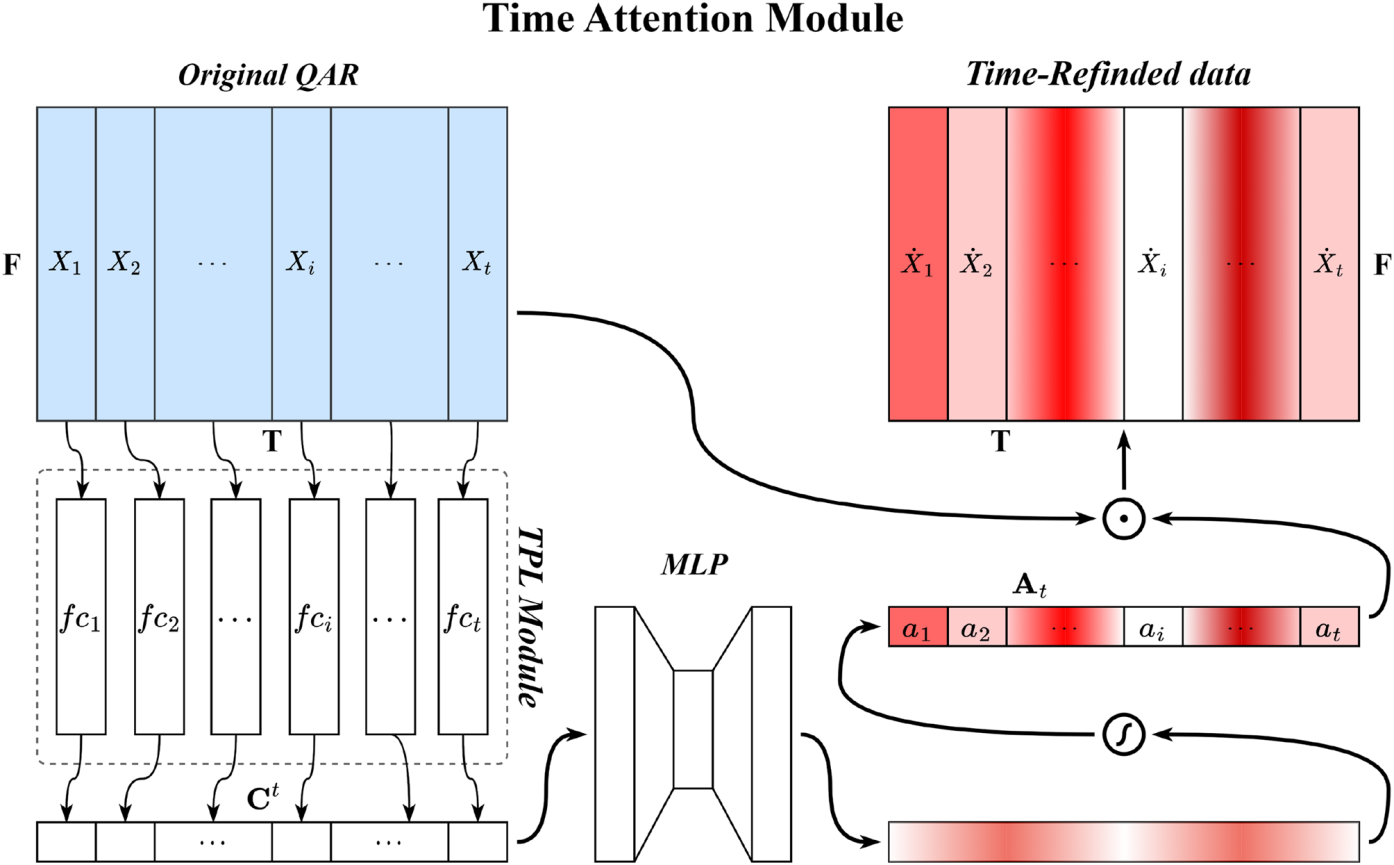
Overview of the time attention module.

**Figure 6:**
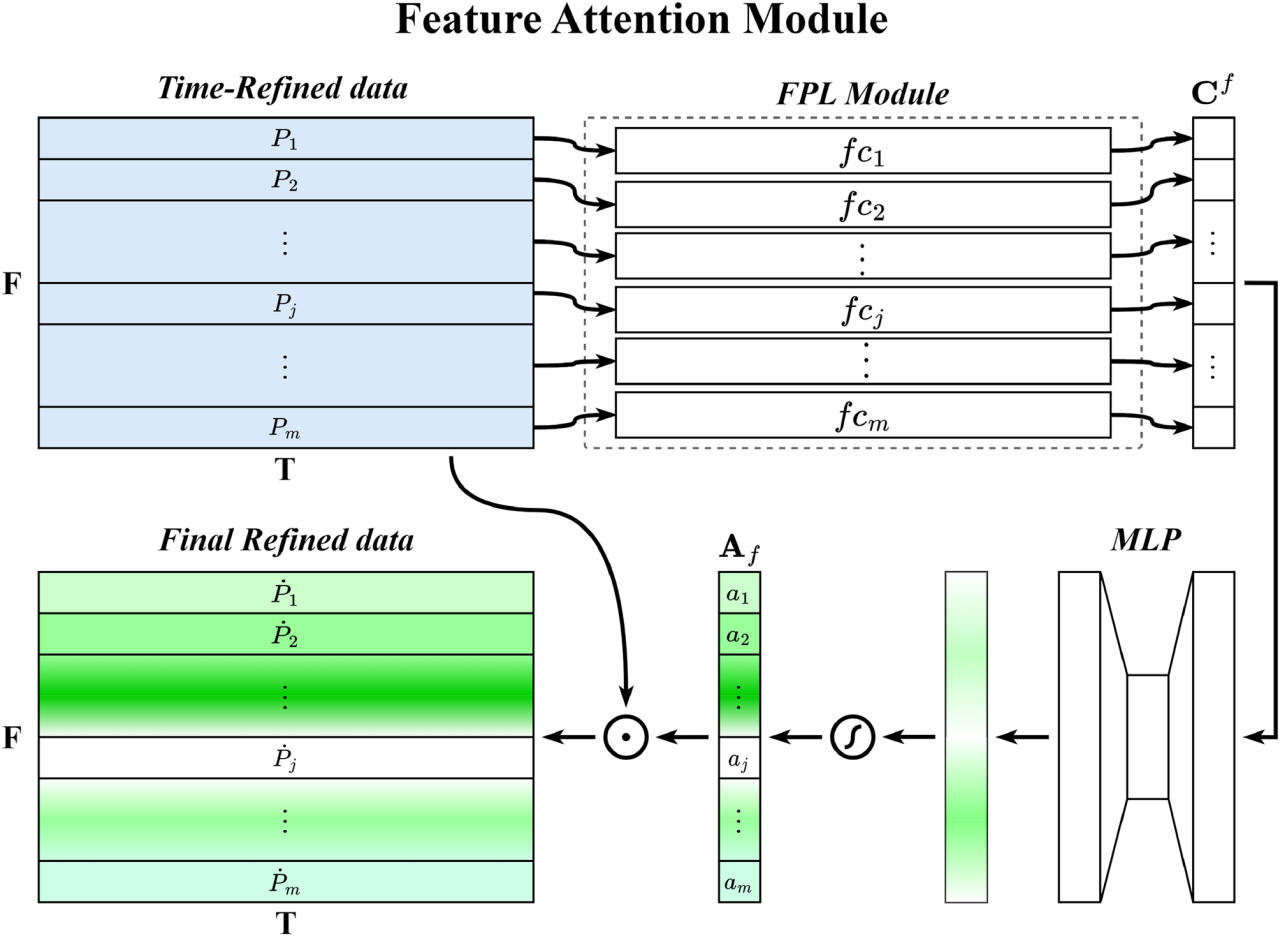
Overview of the feature attention module.

The TFA module contains two submodules, the Time Attention Module (TAM) and the Feature Attention Module (FAM), which are placed together in sequential order. Given an original QAR time series $S\in {\mathbb{R}}^{F\times T}$, a one-dimensional time attention map ${A}_{t}\in {\mathbb{R}}^{1\times T}$ is first produced by TAM and is then multiplied by $S\in {\mathbb{R}}^{F\times T}$ to generate the time-refined data ${S}^{\prime}\in {\mathbb{R}}^{F\times T}$. Immediately afterward, the FAM takes time-refined data ${S}^{\prime}\in {\mathbb{R}}^{F\times T}$ as the input and infers a one-dimensional feature attention map ${A}_{f}\in {\mathbb{R}}^{F\times 1}$, which is immediately multiplied by ${S}^{\prime}\in {\mathbb{R}}^{F\times T}$ to obtain the final refined data $S^{\prime\prime}\in {\mathbb{R}}^{F\times T}$. Figure 4 illustrates the overall computation process of the TFA module, which can be summarized as follows:

1 $$ \begin{aligned} S^{\prime} & = A_{t} (S) \odot S \hfill \\ S^{\prime\prime} & = A_{f} (S^{\prime} ) \odot S^{\prime} \hfill \\ \end{aligned} $$

where $\odot$ stands for the Hadamard product. During multiplication, time attention values are broadcast along the direction of the dimension of flight parameters, while the values of feature attention are broadcast along the direction of the time dimension. Figures 5 and 6 show the overviews of the time attention module and feature attention module, respectively. In the remainder of this section, we will cover the details of these two modules.

- (1) Time attention module (TAM): The role of the time attention module is to highlight the important moments of QAR time series data and suppress the unnecessary moments. Within the time attention module, this is achieved by increasing the representation weight of important flight moments while decreasing the representation weight of unnecessary ones. To produce the attention map, we exploit the relationship between the different flight moments of the QAR data. As each time point of QAR data is considered a time detector, time attention focuses on the time points that are meaningful (‘when’) given input QAR data. The time attention is calculated by collecting and squeezing the information of the feature dimension of QAR data. For this, a network module, namely Time Perceptron List (TPL), is proposed to aggregate the feature information, as shown in Fig. 5. The detailed operation process of the attention module is described below.
  Given original QAR data $S\in {\mathbb{R}}^{F\times T}$ as input, TAM first uses the TPL module to aggregate the information of the feature dimension of $S\in {\mathbb{R}}^{F\times T}$, generating a time context descriptor ${{\varvec{C}}}^{t}=\left\{{c}_{1}^{t},{c}_{2}^{t},\ldots ,{c}_{i}^{t},\ldots ,{c}_{n}^{t}\right\}$. The TPL consists of multiple single-layer perceptions that are arranged in a sequential manner along the time axis. The number of multiple single-layer perceptions is equal to the length of the QAR time series. Each single-layer perceptron ${fc}_{i}$ is used to collect the feature information of the QAR time series at time $i$, generating a context descriptor ${c}_{i}^{t}$. To produce our time attention map ${A}_{t}\in {\mathbb{R}}^{1\times T}$, the time context descriptor is then forwarded to a Multi-Layer Perceptron (MLP) network with one hidden layer. The activation size of the hidden layer is set to ${\mathbb{R}}^{T/r\times 1}$ to reduce the overhead of the model's parameters, and *r* is the reduction ratio. After the time attention map passes through a sigmoid function, it is multiplied with the original QAR time series $S\in {\mathbb{R}}^{F\times T}$ using the Hadamard product, resulting in time-refined data ${S}^{\prime}\in {\mathbb{R}}^{F\times T}$. In short, the time attention is computed as:
  2 $$ \begin{aligned} A_{t} & = \sigma (MLP(TPL(S))) \\ & = \sigma (W_{1}^{MLP} (W_{0}^{MLP} [W_{1}^{TPL} \cdot X_{1} ,W_{2}^{TPL} \cdot X_{2} , \ldots ,W_{i}^{TPL} \cdot X_{i} , \ldots ,W_{t}^{TPL} \cdot X_{t} ])) \\ \end{aligned} $$
  where $\sigma$ stands for the sigmoid function, ${W}_{0}^{MLP}$ and ${W}_{1}^{MLP}$ stand for the weights of the MLP network, and ${W}_{0}^{MLP}$ is followed by a Rectified Linear Unit (ReLU) activation function. ${W}_{i}^{TPL}$ stands for the weight of ${fc}_{i}$ in the TPL.
- (2) Feature attention module (FAM): The role of the feature attention mechanism is to focus on “which” features are informative. It can be considered complementary to time attention, which highlights the important flight feature parameters of time-refined QAR data and suppresses the unnecessary ones. A feature attention map is generated by exploiting the inter-feature relationships of the given QAR data. Each feature series of the QAR data works as a feature detector and is used for calculating its feature attention value by collecting and squeezing its information of the time dimension. Similar to the calculation of time attention, a Feature Perceptron List (FPL) module is constructed to aggregate feature information, as shown in Fig. 6.
  Given time-refined data ${S}^{\prime}\in {\mathbb{R}}^{F\times T}$, we first aggregate the feature information along the time axis of ${S}^{\prime}\in {\mathbb{R}}^{F\times T}$ by using the FPL module, generating a feature context descriptor ${\mathbf{C}}^{f} = \{ c_{1}^{f} ,c_{2}^{f} , \ldots ,c_{j}^{f} , \ldots ,c_{m}^{f} \}$. All the single-layer perceptions are arranged along the feature axis, and each single-layer perceptron ${fc}_{j}$ in the FPL module is used to collect the feature information along the time axis of the $j$ th feature. The feature context descriptor is also then forwarded to a new network composed of an MLP with a hidden layer, producing a feature attention map ${A}_{f}\in {\mathbb{R}}^{F\times 1}$. The activation size of the hidden layer is set to ${\mathbb{R}}^{F/r\times 1}$ to reduce the overhead of the model’s parameters, where *r* is the reduction ratio. After the feature map passes a sigmoid function, it is multiplied with time-refined data ${S}^{\prime}\in {\mathbb{R}}^{F\times T}$ using the Hadamard product, resulting in the final refined data ${S}^{"}\in {\mathbb{R}}^{F\times T}$. In short, the feature attention is computed as:
  3 $$ \begin{aligned} A_{f} & = \sigma (MLP(FPL(S^{\prime} ))) \\ & = \sigma (W_{1}^{MLP} (W_{0}^{MLP} [W_{1}^{FPL} \cdot P_{1} ,W_{2}^{FPL} \cdot P_{2} , \ldots ,W_{i}^{FPL} \cdot P_{i} , \ldots ,W_{f}^{FPL} \cdot P_{f} ])) \\ \end{aligned} $$
  where $\sigma$ stands for the sigmoid function, ${W}_{0}^{MLP}$ and ${W}_{1}^{MLP}$ stand for the weights of the MLP network, and ${W}_{0}^{MLP}$ is followed by a ReLU activation function. ${W}_{j}^{FPL}$ stands for the weight of ${fc}_{j}$ in the FPL.

### TFA-CAE model for QAR feature extraction

AE architectures, including Convolutional Neural Network AEs (CNN-AEs) and Recurrent Neural Network AEs (RNN-AEs), have been demonstrated to be powerful nonlinear feature extraction models, boasting both flexibility and diversity. Typically, the nature of input data determines the selection of model architecture. Previously, it was generally accepted that RNN-based AEs were the preferred choice for dealing with time series data, while CNN-based AEs were preferred for image data. Nevertheless, it has recently been demonstrated that CNN-based AEs outperform general RNN-based AEs on time series data^41^. With complex structures, CNNs are able to extract richer and more complicated hidden features from high-dimensional data than RNNs^42^. Therefore, CNNs are selected to construct our AE model to extract flight features from QAR data.

**Figure 7:**
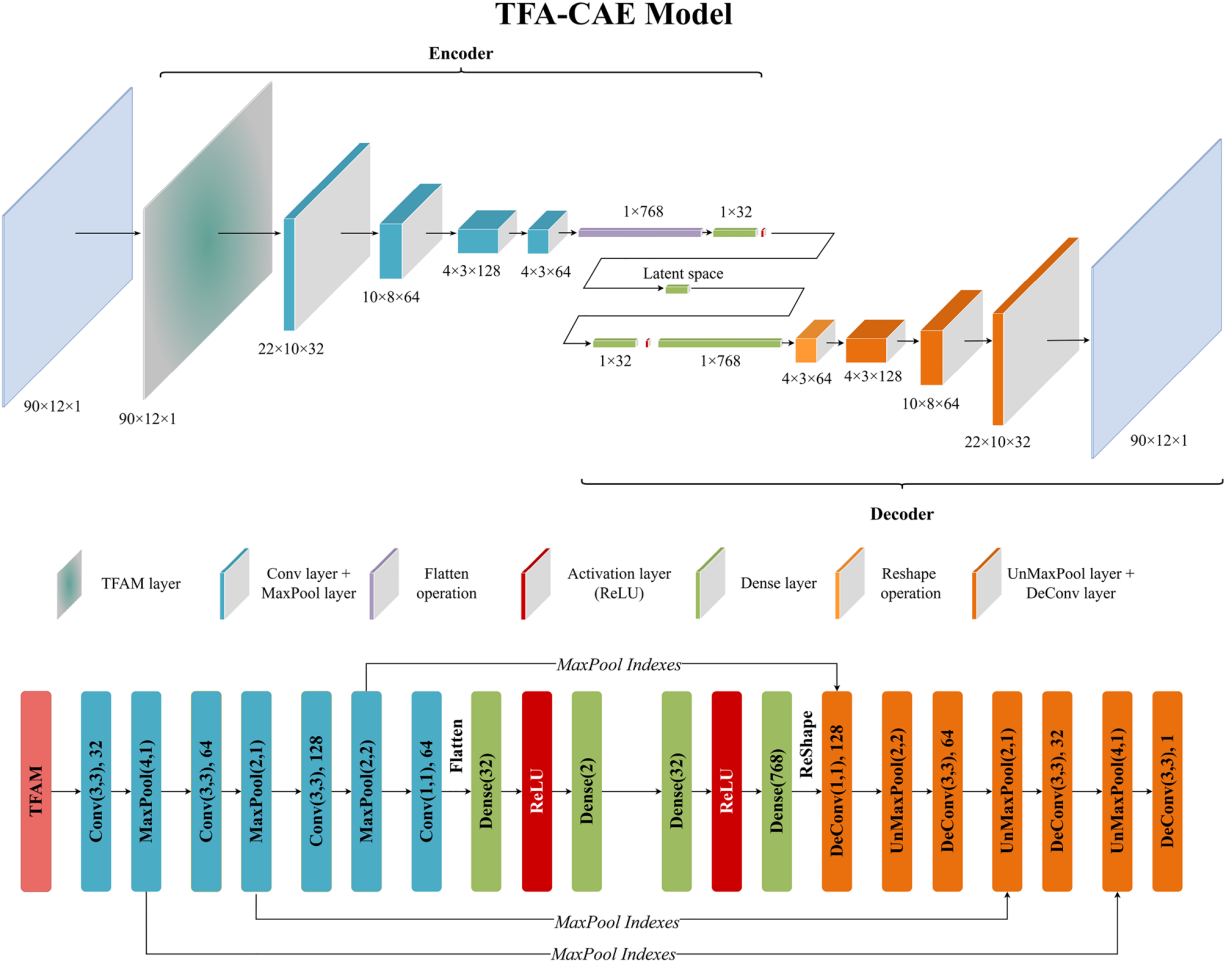
The special structure and parameters of the TFA-CAE model.

In this article, we construct a Time-Feature Attention-based Convolutional Auto-Encoder (TFA-CAE) network model for extracting flight features from QAR time series data. Figure 7 shows the details of the TFA-CAE model, including its special structure and parameters. The TFA-CAE model mainly consists of three parts: the TFA module, an encoder and a decoder, where the TFA module is followed by a CAE. The overall workflow of the TFA-CAE model is described below.

The TFA module is first applied to the original QAR data, producing the final refined time series data. Within the encoder, multiple convolutional layers and max-pooling layers are stacked in an interleaved manner for the extraction of hierarchical features. A 1D vector is generated by flattening all the units within the output of the last convolutional layer and is then transformed into a low-dimensional feature space (latent space) by the two subsequent fully connected layers. Designed as a symmetric form to the encoder, the decoder is composed of multiple unmax-pooling and deconvolutional layers that are stacked in an interleaved manner to reconstruct the original QAR data from the latent features. Moreover, during the training process of the TFA-CAE model, the indices of each max-pool layer within the encoder are fed to the symmetrical unmax-pooling layer within the decoder to perform upsampling. The parameters of the model are optimized through back-propagation of the error loss between the original QAR data and the reconstructed output of the decoder.

## Case study

### Experimental data

In this article, the flight datasets landing at Kunming Changshui International (ICAO: ZPPP, hereafter) and Lhasa Gonggar International (ICAO: ZULS, hereafter) airports are taken as the experimental data for our case study. The dataset contains 12,176 flights, and all the flights are extracted in the way shown in Fig. 3. All flights are sampled with the flight parameters listed in Table 1. After being standardized by min–max normalization, we split the dataset into a training dataset for training the model, a validation dataset to determine when to stop the model training process and a test dataset to evaluate the performance of the model. The dataset is divided in the ratio of 6:2:2. Table 2 presents the details of the division of each dataset.

**Table 2:** The details of the divisions of the three datasets.

Flight phase	Dataset name	Dataset size
Landing	Training dataset	7305
	Validation dataset	2435
	Test dataset	2436

### Model training

Self-attention^43^, as a well-known attention mechanism variant, was proposed with the aim of capturing the internal relationships of data or features and has exhibited great performance in various applications, such as translation. This is similar to the idea of our TFA module proposed in this article. In the experiments of this article, we also construct a Self-Attention-based CAE (SA-CAE) model to extract flight features. In addition, we also adopted traditional CAE, Gate Recurrent Unit based Auto-Encoder (GRU-AE) and TFA-GRU-AE models for comparison with the TFA-CAE model. The PyTorch deep learning framework (version 1.11) is employed to construct and train all the above models. Moreover, the adaptive moment estimation (Adam) optimizer is employed for the optimization of all models. The batch size of the QAR training data is set as 32, and the learning rate is set as 0.0001. During the training processes of all network models, we introduce an early-stopping mechanism to decide when to terminate the training of models. Its patience is set to 15, meaning that the model training process is stopped when the error loss induced on the validation set no longer decreases after 15 epochs. In addition, the reduction ratios as for time attention and feature attention are fixed to 16 and 4, respectively.

Noteworthy, a small fraction of anomalous flights deviated from the common flight pattern in the dataset, which may be due to harsh external atmospheric environments, improper pilot operations and malfunctions of the aircraft themselves. Therefore, to minimize the distortion of these anomalous flights on the model during the training process, we adopt the Huber loss function^44^ with lower anomaly sensitivity to compute the error loss value. The Huber loss function is shown in Eq. (4):

4 $${L}_{\delta }\left(y,f\left(x\right)\right)=\left\{\begin{array}{ll}{\frac{1}{2}(y-f(x))}^{2}, & \quad for \left|y-f\left(x\right)\right|\le \delta \\ \delta \cdot \left(\left|y-f\left(x\right)\right|-\frac{1}{2}\delta \right), & \quad otherwise.\end{array}\right.$$

where $y-f(x)$ is the residual and $\delta$ is the threshold parameter. When the residual is larger than $\delta$, the Huber loss function uses the Mean Absolute Error (MAE) function to calculate the loss error; otherwise, the Mean Squared Error (MSE) function is employed to calculate the loss error. The setting of $\delta$ determines how anomalies are viewed. In the process of model training, each model is trained several times with $\delta$ ranging between 0.1 and 1; the step is 0.1. The setting of $\delta$ is decided when the average loss value on the test data first decreases and remains stable afterward. Eventually, the values of $\delta$ are set to 0.5 for the model.

**Figure 8:**
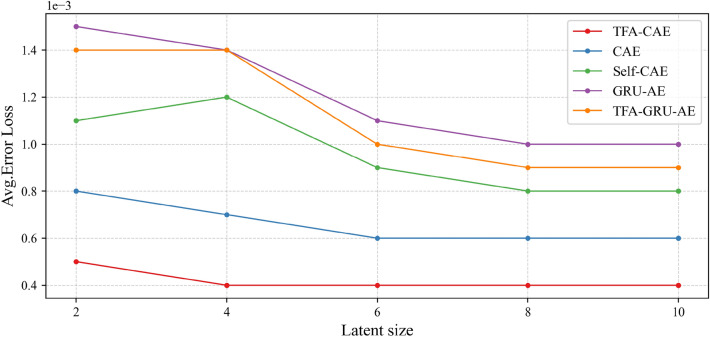
Comparison among the average loss values of the models on the test dataset.

As described in "Introduction" section concerning the AE network model, the AE uses the encoder to map the original input to the feature representation in the latent space and the decoder to reconstruct the original input with the feature representation. Therefore, a smaller error loss indicates a better feature representation from the original QAR data. In this article, all the AE models are trained with multiple dimensions of the latent space. The average loss values of all the models are shown in Fig. 8. By comparing the average loss values of the models, we can first see that the CAE models can extract more representative flight features than the GRU-AE models since the CAE models have smaller average loss values than the GRU-AE models. TFA module helps the models extract more representative flight features. The AE models with TFA module have smaller average loss values than the corresponding ones without TFA module. TFA-CAE model outperforms the other models in terms of flight feature extraction from QAR data since it attains the smallest average loss values as shown in Fig. 8.

### Visualization results of feature extraction

With the latent space size (extracted features) set to 2 in our case study, we can visualize the extracted flight feature results. The visualization outcome of the flight features extracted by the CAE and GRU-AE models and the PCA during the landing phase is shown in Fig. 8. All flight features extracted by each individual model are labeled with their different flight patterns split by the head angle (magnetic north).

**Figure 9:**
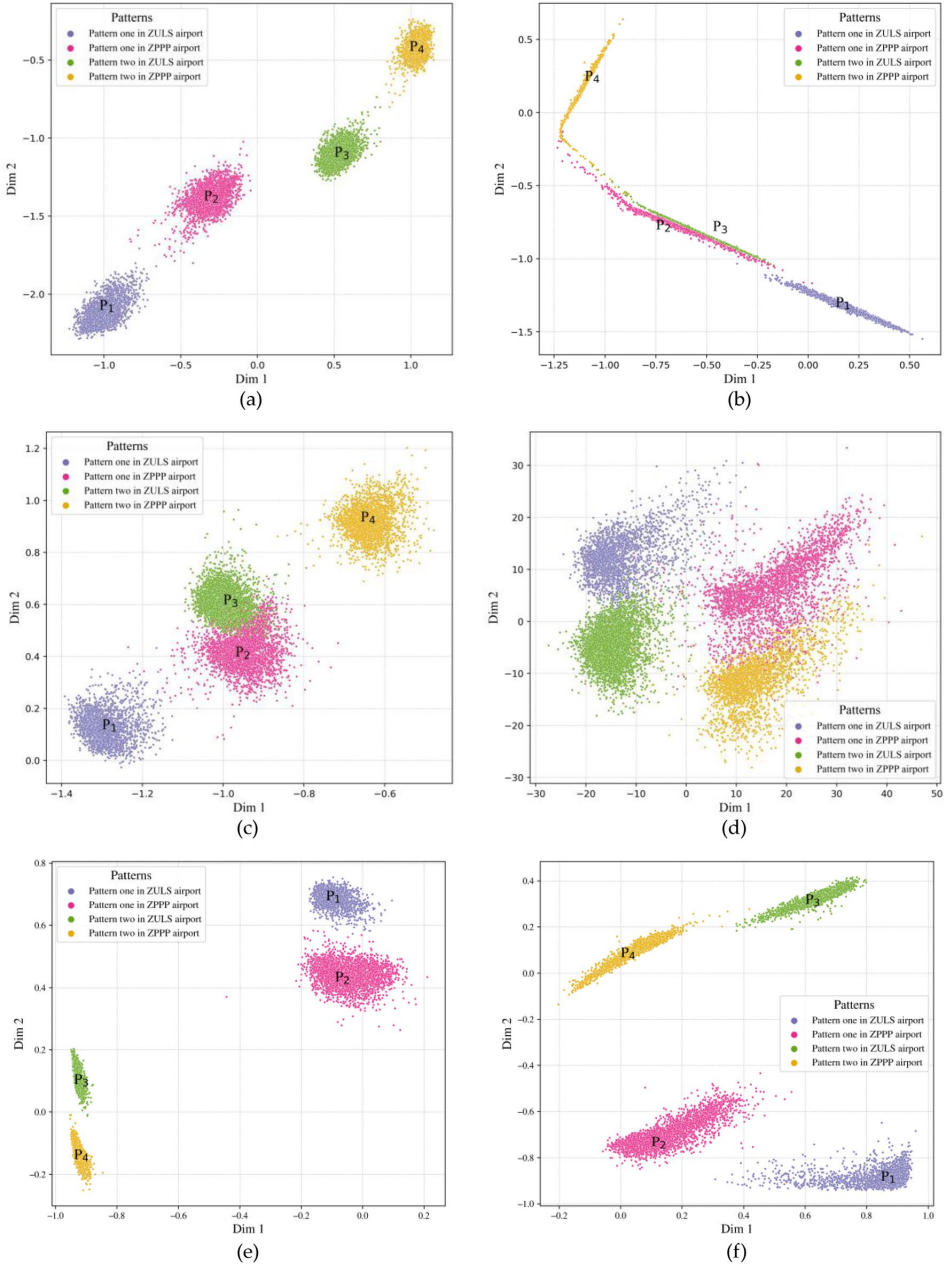
Flight feature extraction results obtained during the ***landing*** phase. As illustrated, subfigure (**a**) is the flight feature result extracted by our TFA-CAE model; (**b**) is the flight feature result extracted by the SA-CAE; (**c**) is the flight feature result extracted by the CAE; (**d**) is the flight feature result extracted by PCA; (**e**) and (**f**) are the flight feature results by the GRU-AE and TFA-GRU-AE respectively.

As the four flight patterns shown in Fig. 9a–f, we can see that both CAE and GRU models outperform the PCA method in discovering flight patterns in the extracted flight features since are not clear in. Moreover, the traditional CAE and SA-CAE models can extract more representative flight features from the original QAR data than GUR-AE and TFA-GRU-AE models. However, the divisions of flight patterns in Fig. 9e and f are much clearer than in Fig. 9b and c, the traditional CAE and SA-CAE models are inferior to GRU-AE and TFA-GRU-AE models in identifying different flight patterns. In addition, TFA module helps both CAE and GRU-AE models to clearly divide the flight patterns by comparing Fig. 9a,f with Fig. 9c,e.

Moreover, as shown in Fig. 9a, the flight objects within the sparse area around each flight pattern are separated clearly and generally considered anomalous flights that deviate from the common flight pattern. Overall, the TFA-CAE model proposed in this article can extract more representative flight features and obtain a better result of the discovery of flight patterns and their division, which provides a well-established technique for further usage of QAR data, such as flight risk detection or FOQA.

### Arrangement of the time and feature attention

**Figure 10:**
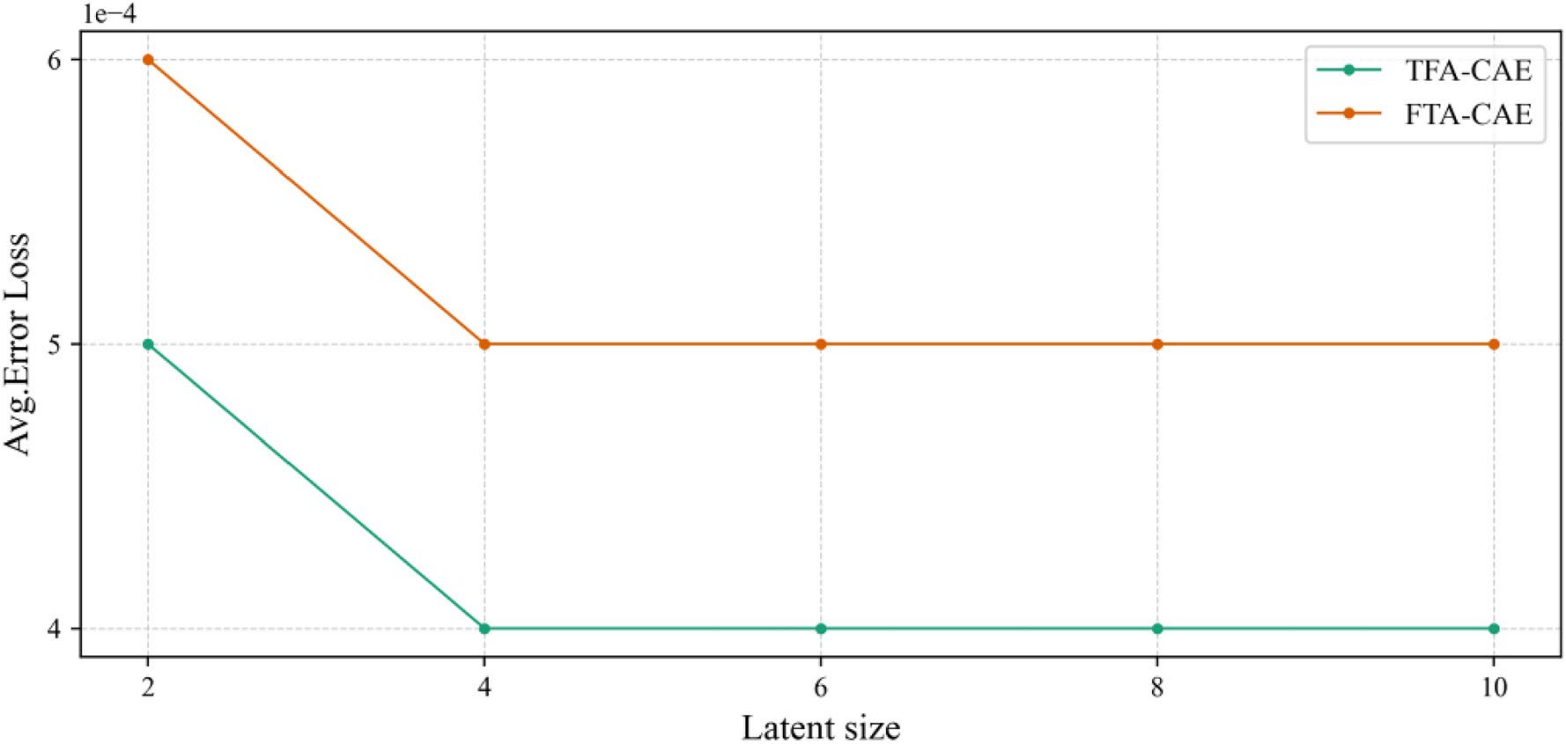
Comparation of the average loss values between TFA-CAE and FTA-CAE models.

For time and feature attention, the arrangement order of these two submodules may affect global performance since each module has different functions. In this section, we compare the two different ways of arranging the time and feature attention submodules: sequential time-feature and sequential feature-time use of both attention modules. A Feature-Time Attention-based CAE (FTA-CAE) model was built and trained to compare with TFA-CAE model. The comparison of the average loss value between TFA-CAE and FTA-CAE models is shown in Fig. 10. From the result, we can see the average loss value of the FTA-CAE model is larger than that of the TFA-CAE model, time-feature attention outperforms feature-time attention in terms of helping the CAE model extract flight features.

**Figure 11:**
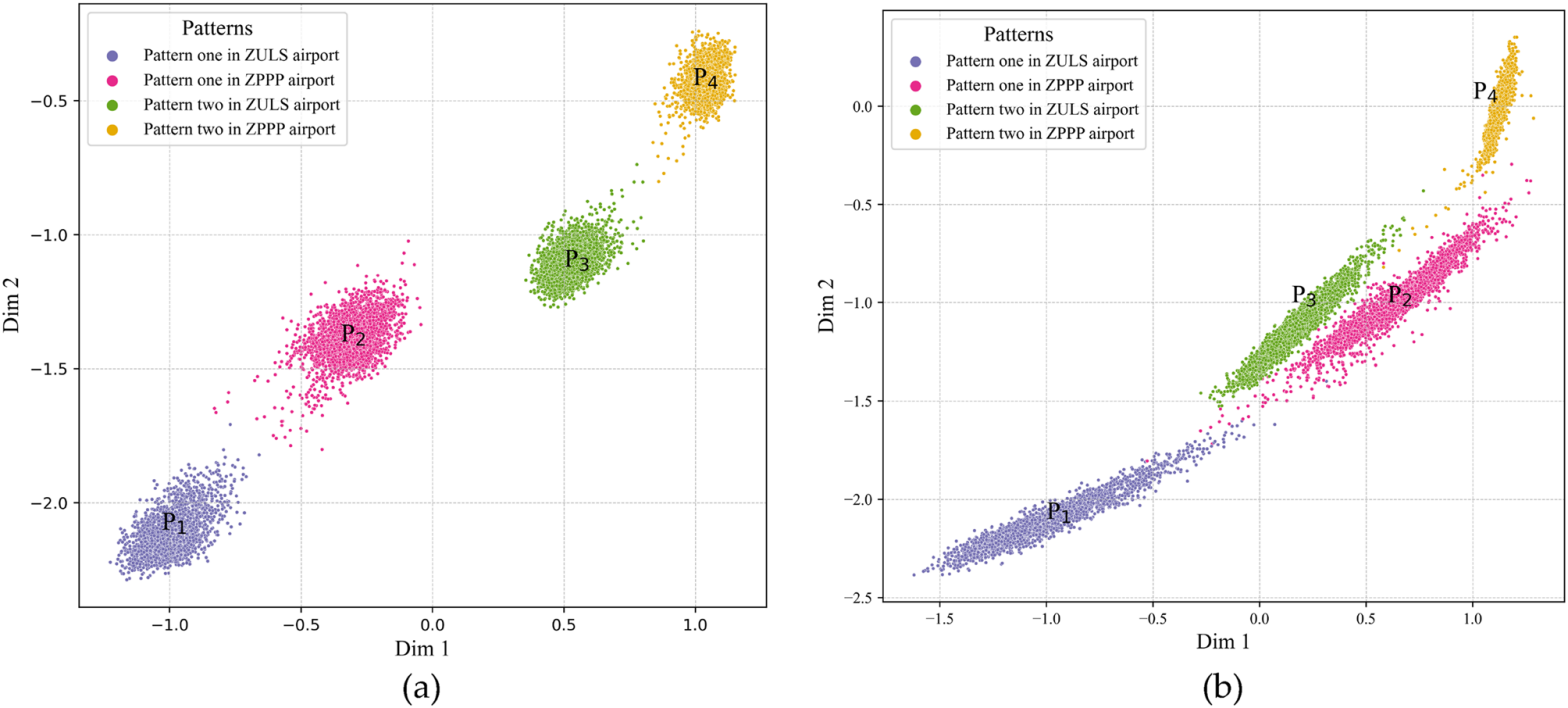
Flight feature extraction results obtained during the ***landing*** phase. As illustrated, subfigure (**a**) is the flight feature result extracted the TFA-CAE model while (**b**) is the flight feature result extracted by the FTA-CAE.

Furthermore, the visualization results of flight features extracted by the FTA-CAE and TFA-CAE models are compared in Fig. 11. As shown in Fig. 11b, the FTA-CAE model is able to discover the four flight patterns within the extracted flight features. By comparing Fig. 11a and b, the TFA-CAE model outperforms the FTA-CAE model in terms of the division of flight patterns since the flight patterns ${P}_{2}$ and ${P}_{3}$ are not clearly divided in Fig. 11b. However, the FTA-CAE model outperforms the SA-CAE and CAE models in the division of flight patterns; the division of these four flight patterns in Fig. 11b is clearer than in both Fig. 9b and c.

Throughout the above comparisons, we can learn that (1) the combination of time and feature attentions can help the CAE model extract more representative flight features from QAR data and (2) the sequential time-feature attention module is better than the sequential feature-time attention module. Our final attention module and TFA-CAE model are shown in Figs. 4 and 7, respectively.

## Conclusion and discussion

In this article, to address the difficulties of mining QAR data caused by their high-dimensional and high-frequency characteristics, we propose a TFA-CAE network model to perform flight feature extraction by essentially capturing the internal relationships among different flight phases as well as different sets of flight parameters. For comparison, the classic PCA approach, traditional CAE network, an SA-CAE and GRU-AE network models were also conducted with the same QAR dataset. The results show that our TFA-CAE model can extract more representative flight features and simultaneously discover runway-level flight patterns that are clearly separated from each other. Moreover, within the extracted flight features, the anomalous flights deviating from the common flight pattern are clearly separated from their corresponding flight patterns. The TFA-CAE model provides a well-established technique for further usage of QAR data, such as flight risk detection or FOQA.

Air transport is playing an increasingly popular and irreplaceable role in transportation, and flight safety has always been a crucial focus in civil aviation safety management. With the expectation that more flights will depart in the future, flight safety management is facing increasing and new challenges. To address these challenges and further enhance flight safety, a shift has been made in civil aviation safety management from post-accident investigation and analysis to pre-accident warning. In response to such a requirement, civil aviation endeavors to effectively prevent potential flight accidents before they occur by innovatively and proactively identifying operationally significant safety events that are currently untracked. By appropriately dealing with these potential aviation safety incidents, the accident rate per year will remain at its lowest historical level. QAR data will provide an effective way to achieve Flight Operation Quality Assurance (FOQA). Since QAR data are onboard-recorded flight data and record many various types of flight parameters, they reflect various real flight situations that occur during the flight process, with factors such as the pilot’s actual basic capabilities and skills, the actual flight patterns, the performance of the aircraft itself and the potential flight faults or anomalies. Massive and rich flight big data provide a complete database for studying flight risks and deep learning methods. With the continuous development of ANNs, the combination of big QAR data and deep learning will provide an important and effective method for flight safety management.

However, we only tried a two-dimensional time-series data set as the input of TFA-CAE model, which could be challengeable when more complex data are provided. Therefore, a more generic technical architecture for extracting flight features from variable-length time series data could be anticipated in the future. Besides, the evaluation is limited to a simple case study with QAR data collected from two specific airports, further experiments and comparisons with more datasets and baseline techniques are required for the generalization and perfection of techniques proposed in this study. In addition, the automatic discovery of common flight patterns and detection of anomalous flights or risks are two future topics that can enable better-targeted flight safety management.

## Data Availability

Data that support the findings of this study are available upon reasonable request to the corresponding author.
